# Wastewater-based tracing of doping use by the general population and amateur athletes

**DOI:** 10.1007/s00216-017-0835-3

**Published:** 2018-01-15

**Authors:** Ana Causanilles, Vera Nordmann, Dennis Vughs, Erik Emke, Olivier de Hon, Félix Hernández, Pim de Voogt

**Affiliations:** 1 KWR Watercycle Research Institute, P.O. Box 1072, 3430 BB Nieuwegein, The Netherlands; 2 Institute for Biodiversity and Ecosystem Dynamics, University of Amsterdam, P.O. Box 94248, 1090 GE Amsterdam, The Netherlands; 3 Anti-Doping Authority The Netherlands, P.O. Box 5000, 2900 EA Capelle aan de IJssel, The Netherlands; 4 Research Institute for Pesticides and Water, University Jaume I, Avda. Sos Baynat s/n, 12071 Castellón, Spain

**Keywords:** Sewage, Doping, Prohibited substances, Sport, Wastewater-based epidemiology

## Abstract

The present study investigates the applicability of the chemical analysis of wastewater to assess the use of doping substances by the general population and amateur athletes. To this end, an analytical methodology that can identify and quantify a list of 15 substances from the groups of anabolic steroids, weight loss products, and masking agents in wastewater has been developed. The method uses solid phase extraction to increase the detection sensitivity of the target analytes, expected to be present at very low concentrations (ng L^−1^ range), and decrease possible matrix interferences. Instrumental analysis is performed by liquid chromatography coupled to high-resolution mass spectrometry, allowing data acquisition in both full scan and tandem MS mode. The method has been successfully validated at two concentration levels (50 and 200 ng L^−1^) with limits of quantification ranging between 0.7 and 60 ng L^−1^, intra- and inter-day precision expressed as relative standard deviation below 15%, procedural recoveries between 60 and 160% and matrix effects ranging from 45 to 121%. The stability of the analytes in wastewater was evaluated at different storage temperatures illustrating the importance of freezing the samples immediately after collection. The application of the method to 24-h composite wastewater samples collected at the entrance of three wastewater treatment plants and one pumping station while different sport events were taking place revealed the presence in wastewater, and hence the use, of the weight loss substances ephedrine, norephedrine, methylhexanamine, and 2,4-dinitrophenol. The use of these stimulants was visible just prior and during the event days and in greater amounts than anabolic steroids or masking agents.

**Electronic supplementary material:**

The online version of this article (10.1007/s00216-017-0835-3) contains supplementary material, which is available to authorized users.

**Graphical abstract:**
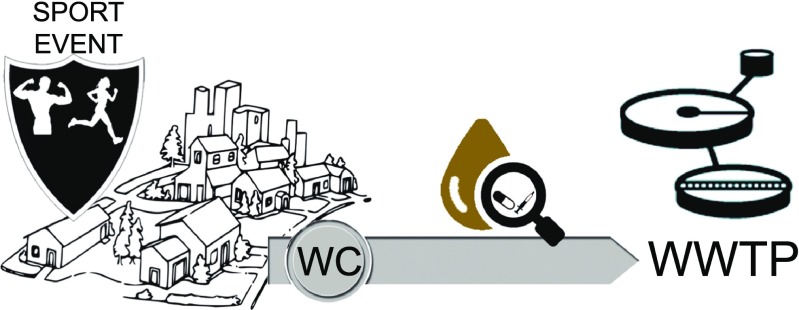
Chemical analysis of untreated wastewater reveals the use of prohibited doping substances during amateur sport event

## Introduction

The prevalence of doping in sports and fitness is an issue of current concern for a healthy society, and in particular for all those involved in sports, for example for evaluating anti-doping policy measures [1–3]. Its evaluation for the general population, however, is not an easy matter, and even though it is virtually impossible to uncover the exact prevalence of a prohibited activity such as doping, various methods are available to expose parts of this particular problem. These include (i) laboratory-based chemical analyses (in biological matrices such as blood and urine), (ii) questionnaires, (iii) inferences from performances, and (iv) inferences from ego documents. Doping control is widely implemented at professional level (e.g., Olympic games and world championships) and advanced standardized methodologies already exist to detect a great variety of prohibited and controlled substances. However, data are scarce and few scientific articles have addressed the subject so far, with results showing a wide variance in the prevalence of doping at both amateur and professional level. For example, a study showed a prevalence of 2.6% among tertiary education students in Europe [3], evaluated by anonymous questionnaires, and up to 8.2% in fitness centers in the Netherlands [2], evaluated by randomized response technique. In the case of elite sports, the range is even larger and likely to be between 14 and 39% [1].

Recently, analytical chemical methods have been developed that may play a more important role in this topic. Wastewater contains the excreted biomarkers of human metabolism that directly reflect the exposure and stressors placed upon an entire contributing community. The quantitative measurement of these specific biomarkers in wastewater from communities allows the averaged patterns of factors related to lifestyle, disease, and environment to be used for the assessment of community health [4]. It has been shown that individual communities present different patterns with respect to the levels of various illicit drug residues in wastewater [5–7] and it is hypothesized that this will also concur for other biomarkers [4]. This approach, known as wastewater-based epidemiology (WBE), has been also used to determine the use of other substances such as erectile dysfunction pharmaceuticals [8], alcohol [9], nicotine, and caffeine [10] and the exposure of a population to pesticides [11] or phthalate plasticizers [12]. Hence, there is the clear potential to develop a wider range of innovative solutions to quantitatively assess patterns of factors related to health, fitness, and illness within populations, while also providing means of collecting data for epidemiological and socio-economic studies.

Professional athletes are tested by national and international organizations on the use of doping substances. Information on the exact amounts of doping substances that are actually being taken is mostly unknown. The same holds true for athletes visiting fitness centers. WBE can be a valuable complimentary approach for national doping authorities, as suggested by recent studies [1, 13]. It can give information on the usage of known compounds in a specified area or group, albeit at the (averaged) group level. The presence of substances that are commonly used either as doping/lifestyle drugs or for the treatment of diverse diseases, has already been identified in wastewaters from fitness centers and wastewater treatment plant (WWTP) influents and effluents [14], providing a deeper insight into the origin and whereabouts of these compounds.

The present study was conducted to further investigate the application of the WBE approach for assessing the use of doping substances, targeting amateur athletes and other users in the general population at specific sport events. To this aim, a sensitive analytical methodology based on solid phase extraction (SPE) followed by liquid chromatography coupled to high-resolution mass spectrometry was developed and validated. The method was applied to 24-h composite wastewater samples collected at the entrance of three WWTPs and one pumping station while different sport events were taking place within the corresponding sewer catchment area.

## Materials and methods

### Reagents and standards

The analytical standards included in the study were as follows: metandienone, metenolone, mibolerone, nandrolone, sibutramine, clomiphene, tamoxifen and anastrozole, obtained from TRC Toronto Research Chemicals Inc. (Ontario, Canada); ephedrine, norephedrine, trenbolone, clenbuterol, methylhexanamine, 2,4-dinitrophenol, and finasteride, obtained from Sigma-Aldrich (Stenheim, Germany). The chemical properties and structures of the analytes selected are compiled in Table S1 (see the Electronic Supplementary Material, ESM). The isotopically labeled internal standards (ILIS) used as surrogates for the quantification of their analogue native analytes were the following: methandrostenolone-d_3_, mibolerone-d_3_, trenbolone-d_5_, sibutramine-d_6_, 2,4-dinitrophenol-d_3_, clomiphene-d_5_, tamoxifen-d_5_, anastrozole-d_12_, and finasteride-d_9_, purchased from TRC; ephedrine-d_3_, norephedrine-d_3_, clenbuterol-d_9_, and methylhexanamine-d_4_, purchased from Sigma-Aldrich. The purity of the ILIS was verified (> 99%) by means of the injection of increasing concentrations up to 160 μg L^−1^, the result being that even in high concentrations no trace of the native signal was detected. In addition, the deuterated standard atrazine-d_5_ was added as external standard to all vials (calibration and samples) prior to analysis in order to monitor the injection and ionization process.

Individual stock solutions were prepared from either the powdered substance or a 1 mg mL^−1^ ampoule solution, in methanol, at a level ranging from 60 to 160 mg L^−1^ for the native compounds and 20 mg L^−1^ for the ILIS, and stored at − 20 °C. Two mix stock solutions containing the native analytical standards and the ILIS separately were prepared at a final level of 1 mg L^−1^ in methanol. Working solutions were prepared by dilution to the desired concentration with methanol and stored at − 20 °C. Calibration curves were prepared daily by diluting with ultrapure water the appropriate mix (containing atrazine-d_5_ as external standard) to a final water/methanol (90:10, *v*/*v*) composition.

Methanol and acetonitrile HPLC-grade solvents, ammonium hydroxide solution (28–30%), and hydrochloric acid were supplied by Avantor Performance Materials B.V (Deventer, The Netherlands). Formic acid, FA (50% in water) was obtained from Fluka Analytical (Sigma-Aldrich, Stenheim, Germany). The ultrapure water was obtained by purifying demineralized water in an Elga Purelab Chorus ultrapure water system (High Wycombe, UK).

Glass fiber filters (type A/E, 1 μm) were purchased from Pall Corporation (Port Washington, NY, USA). SPE cartridges, built of a mixed-mode, reversed-phase/strong cation exchange, water-wettable polymer (Oasis MCX, 150 mg, 6 cm^3^) were obtained from Waters (Milford, MA, USA).

For the calibration of the Sciex TripleTOF mass spectrometer APCI positive and negative calibration solutions were obtained from AB Sciex (MA, US).

### Sample collection

Four 15-day sampling campaigns were carried out at four different locations, targeting three different sport events. The 2-week sampling period was planned including samples from a period where no sport event was taking place to determine the background concentrations corresponding to excretions from the regular population. This allowed the comparison to the event period, where visitors and athletes would add up. The samples collected during the four sampling campaigns were stored in HDPE bottles at − 20 °C until analysis.

The targeted sport events and the sampling strategy are described below:

#### Event A

This relatively large 5-day Olympic sport event took place in a large size city (between 500,000 and 1000,000 inhabitants). More than 1000 professional athletes participated, all of who were monitored by the international anti-doping system in place as overseen by the World Anti-Doping Agency. In addition, 125,000 visitor tickets were sold to attend the different events. The sample collection was performed at the entrance of the WWTP, at a 10-km distance from the main event location. The 24-h composite samples were collected in volume proportional mode, with an average sampling frequency of 3.5 min.

#### Event B

The second was a relatively small 1-day bodybuilding event that took place in a small-size city (less than 100,000 inhabitants). It gathered 500 amateur athletes, coaches and volunteers, and more than 800 visitors. There were no anti-doping controls in place for this event. The sample collection was performed at the entrance of the WWTP, at a 3-km distance from the event location. The 24-h composite samples were collected in volume proportional mode, with an average sampling frequency of 12 min.

#### Event C

The third was a relatively large 2-day bodybuilding event that took place in a town (less than 100,000 inhabitants) close to a medium-size city (between 100,000 and 500,000 inhabitants). Over 100 amateur athletes participated and 8000 visitors attended. There were no anti-doping controls in place for this event. The sample collection was performed at two locations, at a pumping station closer to the source (in the town) and at the entrance of the WWTP serving the larger catchment area (that included both the town and the medium-size city). The distance from the event location to the sampling collection points were 5 and 12 km, respectively. At the pumping station, the composite samples were collected in time-proportional mode with a frequency of 5 min, whereas at the WWTP they were collected in volume proportional mode, with an average sampling frequency of 12 min.

### Analytical methodology

#### Sample treatment

Fifty milliliters of homogenized sample were spiked at 200 ng L^−1^ with ILIS to act as surrogates during the sample handling and analysis and to correct for possible analyte losses and/or matrix effects. The samples were filtered using a 1-μm type A/E glass fiber filter and acidified to pH 2 to 3 with a solution of hydrochloric acid. Next, the sample was loaded onto a mix-mode cationic polymer-based cartridge (Oasis MCX) previously conditioned with 8 mL of methanol and 8 mL of ultrapure water acidified with 2% FA. After loading the samples, the cartridges were washed with 4 mL of ultrapure water acidified with 2% FA. Next, the cartridges were vacuum dried. Prior to elution, a second washing step with 4 mL of ultrapure water with 5% acetonitrile acidified with 2% FA was performed. Elution was done in two steps, using 4 mL of acetonitrile followed by 4 mL of acetonitrile with 5% NH_4_OH, both collected as one eluate. The eluate was evaporated to 250 μL by means of a Barkey optocontrol (Germany) with a gentle N_2_ stream (block temperature set at 300 °C), where after 250 μL of ultrapure water were added and evaporated again to 250 μL (adjusted by weight) and reconstituted to 0.5 mL of water/methanol 90:10 (*v*/*v*) with a 80:20 solution containing atrazine-d_5_ as external standard.

#### Instrumental analysis

Fifty milliliters of the sample extracts were injected into a UPLC (NEXERA X2 LC-30AD, Shimadzu Corporation, Kyoto, Japan) coupled to a time of flight high-resolution mass spectrometer (TripleTOF 5600+, AB Sciex, MA, US). The LC separation was performed on a XBridge BEH XP C18 column (Waters) with particle size 2.5 μm, and dimensions of 2.1 mm × 150 mm, preceded by a 2.0 mm × 2.1 mm I.D. Phenomenex SecurityGuard Ultra column (Phenomenex, Torrance, USA), at a constant flow rate of 0.250 mL min^−1^. Mobile phase solvents were ultrapure water and methanol both with 0.05% FA (*v*/*v*). The percentage of organic solvent changed as follows: 0 min, 20%; 12 min, 100%; 15 min, 100%; 16 min, 20%; 20 min, 20%. Between consecutive runs, the analytical column was re-equilibrated for 4 min.

The TripleTOF was set up to acquire the full scan in the range of 50 to 800 *m/z* as well as the full scan of the product ions of the target compounds, for which the retention time and optimal collision energy were pre-set. Mass calibration was performed with every batch run just prior to the sequence start. The system operated in positive ionization mode, with the ion spray voltage at 5 kV, source temperature at 500 °C and declustering potential adjusted to 70 V. For 2,4-dinitrophenol, the system was operated in negative mode, with the ion spray voltage at − 4.5 kV, source temperature at 500 °C and declustering potential adjusted to − 70 V. Gas 1, gas 2, and curtain gas were set at 40, 50, and 25 psi, respectively.

Data processing was performed with the MultiQuant 3.0 software, version 3.0.5373.0 (AB Sciex). Analyte concentrations were quantified from the sum of the acquired product ions relative signal (native divided by the corresponding deuterated analogue, when available; when it was not available, a closely resembling deuterated was selected). The acquisition and data processing parameters can be found in Table S2 (see the ESM). In addition, GraphPad Prism 5 was used for post statistical evaluation of the results.

Analyte concentrations were multiplied with 24-h flow rates to obtain daily loads. For comparison between different cities, daily loads were normalized to the numbers of inhabitants connected to the corresponding sewer system that were obtained from census data provided by the WWTP managers (see ESM Table S3). The number of inhabitants used for the calculation was not corrected for the number of extra people attending each event, since the additional number did not change the normalized load with more than 1%, and we assumed that many visitors would be the people living in the cities, and therefore already accounted in the number of inhabitants.

#### Method validation

The method was validated in terms of linearity, limits of detection and quantification, precision intra-day and inter-day (repeatability), procedural recovery (accuracy), and matrix effects by analyzing wastewater spiked with selected analytes.

A calibration curve was established by analyzing spiked ultrapure water and wastewater with standard solutions at 10 different concentrations, ranging from 0 to 50 μg L^−1^, to investigate linearity. Limits of detection and quantification (LOD and LOQ, respectively) were defined as the concentration that provides signal-to-noise (S/N) values of 3 and 10 for the quantifier ion of each analyte. The values were calculated at the lowest point of the calibration curve. Intra-day and inter-day precision was assessed at two levels, 50 and 200 ng L^−1^, with six replicates per level, and during three non-consecutive days. Procedural recovery (%) was calculated as the ratio of the signal of the analyte spiked to a sample after sample treatment (*γ*) against the signal of the analyte spiked to the same sample before treatment (*β*): [*γ*/*β*] × 100. Matrix effect (%) was calculated as the ratio of the signal of solution *β* (where the signal of the native compound present in the used sample was subtracted) against the signal of the analyte spiked to ultrapure water (*α*): [*β*/*α*] × 100.

#### Analyte stability

It was important to assess the stability of the target compounds in wastewater, in-sewer and in-sample conditions, since the samples were stored at − 20 °C between 1 and 8 months before analysis. Besides, samples were kept in the autosampler at 4 °C during the 24-h cycle. In addition, in the case of the automated sampling at the pumping station (event C), there was sometimes a delay and samples had to be left longer in the autosampler (max 24 h).

To evaluate the stability, wastewater samples were spiked at 400 ng L^−1^ in order to assure quantification of the parent compound, even if it would degrade by tenfold. The stability test was performed in triplicate for each temperature and storage condition. The experiment was scheduled to last a month and a half, with six sampling points during that period, and using three different storage temperature conditions: freezer (− 20 °C), fridge (4 °C), and laboratory room temperature (20 °C). Samples were prepared and stored in polypropylene tubes.

This type of experiment allows measuring the total degradation that compounds might suffer in wastewater due to transformation processes in-sewer and storage; however, it is neither possible to differentiate the type of degradation (chemical, biological, or physical) nor to identify when (during in-sewer transport or storage) the degradation may have occurred [15–17].

## Results and discussion

### Selection of target compounds

The choice of substances was made in order to detect compounds relevant for actual doping use, such as those promoting muscle growth (anabolic steroids), increasing metabolism by burning fat (weight loss stimulants), or hiding the derived effects or preventing the detection of doping substances use (masking agents). Table S1 (see the ESM) presents the list of compounds, which it is not all-inclusive but fit-for-purpose for this research, with a description of their type of action and licit formulation (ATC code) when available. Not all of them are mentioned on the 2017 Prohibited List as published by the World Anti-Doping Agency or known to be abused in a fitness-setting. For example, finasteride is not prohibited but mentioned as a confounding factor used to alter athlete’s steroid profile and 2,4-dinitrophenol, also not listed but considered relevant due to its use being associated with fatal incidents [18].

### Method development

#### Sample treatment

Sample preparation plays an important role in the method development because wastewater is a very complex matrix and target analytes are expected at the low ng L^−1^ concentration level. Preparatory steps such as sample dilution and filtration to minimize matrix interferences as well as the different parameters involved in the concentration by SPE required optimization. For the optimization of the sample treatment, a pooled wastewater reference sample was used as matrix.

First, the effect of diluting the sample was investigated at four dilution levels in triplicate (1× (no dilution), 2×, 5×, 10×). Results showed no impact on the overall recoveries (defined as the comparison between the quantified concentration and the nominal spike value); therefore, dilution of the sample was not performed during the method development. Second, the effect of sample filtration was evaluated for four different filter materials: type GF/F glass microfiber filter (0.7 μm, *Ø* 47 mm, Whatman), regenerated cellulose (0.2 μm, *Ø* 47 mm, Satorius), type A/E glass fiber filter (1 μm, *Ø* 47 mm, Pall), and polyethersulfone (PES) membrane (0.2 μm, *Ø* 90 mm, Nalgene). Removing the particulate phase from the sample by filtering could have implications for the calculation of the daily loads for compounds having a high log *K*_ow_ (or *K*_oc_). For the compounds observed at levels above their LOQ, this holds only for sibutramine and metandienone. Their fraction sorbed could be substantial if the particle concentration would be more than 1 g L^−1^. We estimated/observed particulate phase concentrations in the samples invariably below 1 g L^−1^ and therefore the contribution of the compounds sorbed to the particle phase negligible. Type A/E glass fiber presented the most convenient combination of good recovery and being less affected by matrix effects and was therefore chosen.

Optimization of the SPE procedure was done with the aim of reaching good extraction recoveries for all the target analytes despite differences in physicochemical properties, and concentrating expected influent concentrations from low ng L^−1^ to the μg L^−1^ range. The pKa of the analytes indicated that the majority of species would be either neutral or positively charged at pH = 7 (see ESM Table S1), except for 2,4-dinitrophenol which has pKa 4.09 and will therefore be almost entirely (89–100%) in its anionic form at pH 5–9. SPE cartridges Oasis HLB and MCX were selected for the optimization test because of their hydrophilic–lipophilic balance and cation exchange properties, respectively. In addition, C18 cartridges were included as they are commonly used for the analysis of doping substances in human urine. Our results showed that weight loss agents as well as anabolic steroids were hardly or not recovered with C18 cartridges. As a result, the use of the C18 sorbent was renounced. Regarding HLB and MCX, they provided satisfactory results overall, but MCX was selected due to its mixed-mode properties that allowed a better retention of weak bases as well as neutral compounds.

Once the MCX cartridge was chosen, the standard procedure recommended by the manufacturer was adjusted by optimizing the solvent composition used for the elution, evaluating the possibility of dividing the elution into two different steps, and adjusting the solvent volumes. The use of acetonitrile rather than methanol decreased the matrix interferences in the eluate, and adding 5% of NH_4_OH allowed higher recovery for those compounds with lower pKa (namely ephedrines). A two-step elution was then selected, with first 4 mL of acetonitrile (best for anabolic steroids) followed by 4 mL of 5% NH4OH in acetonitrile, combining these to a final eluate of 8 mL.

Finally, the effect of washing was investigated, including up to two steps, one before and/or one after the cartridge drying, with different combinations of ultrapure water and/or 2% FA and/or 5% acetonitrile. Results for anabolic steroids and masking agents improved when a washing step after drying the cartridge was incorporated. Therefore, the final procedure consisted of washing with 2% FA in ultrapure water before drying and another washing step with 5% acetonitrile and 2% FA in ultrapure water after drying.

#### LC-MS/MS (HRMS)

The sensitivity and selectivity of the analytical method was optimized by selecting the most appropriate LC column, the mobile phase composition and gradient, and the injection volume (for the LC part) and the product ions (for the MS part).

Three LC columns were tested: Xbridge C18 150 × 2.1 mm, 2.5 μm particle size, Kinetex 1.7 μm F5 100 A 150 × 2.1 mm, and Kinetex 1.7 μm Biphenyl 100 A 150 × 2.1 mm. The column selected was the Xbridge because it provided sufficient separation for the target compounds and its characteristics were suitable for a wide range of compounds in the case that a screening for suspects was applied. Both, acetonitrile and methanol with 0.05% FA were tested as organic solvent for the mobile phase composition. Acetonitrile provided lower backpressure in the LC column and better peak shape and sensitivity for methylhexanamine. However, the peak shape for three masking agents worsened, and clomiphene isomers were not resolved. To compromise the general peak shape methanol was selected, and to control the backpressure the flow was adjusted to 0.25 mL min^−1^. The mobile phase gradient was important because all anabolic steroids were eluting within the 10–13 min range. Modifications of the gradient were tested to improve the separation, but no clear improvements were observed while the run time increased substantially. In addition, the injection volume was set to 50 μL as a compromise between a higher signal while keeping a Gaussian peak shape. For the MS part, individual analytes were injected in order to obtain the optimal collision energy and the accurate mass of at least one product ion in addition to the accurate mass of the protonated ion. As can be seen in Table S2 (see the ESM), two transitions were acquired for all compounds, with the only exception of methylhexanamine and 2,4-dinitrophenol, for which only one product ion was used, due to the small molecule size, which made it troublesome to obtain more specific ions and/or with enough sensitivity.

#### Analyte stability

A summary of the results of the stability tests is presented in Table 1, which shows the time points at which the change in concentration compared to the initial concentration is larger than 20%, for each compound and temperature. Figure S1 (see the ESM) presents the full data evaluation. In order to identify outlying values, for each compound, a stability model was generated based on a quadratic or linear fit, as has been previously done for other illicit substances [19, 20]. The statistical evaluation revealed that a linear fit was the preferred model in most cases except for sibutramine at 20 °C, trenbolone at − 20 °C, metandienone at 20 °C, and clomiphene at 4 and − 20 °C, where a quadratic fit was preferred. Outliers were identified by the best-fit model evaluation and excluded from the graphs. A variation of ± 20% was not considered significant as it was accepted as the method variability. Our results highlight the importance of immediately storing the samples in the freezer after collection (− 20 °C); otherwise, target compounds might undergo significant degradation. At − 20 °C, only for clomiphene a loss of 25% after 3 days was observed, which could be related to its low solubility in water. When stored at 4 °C, most analytes remained stable in the studied period; however, compounds such as metandienone experienced 50% decrease after 7 days, and metenolone, tamoxifen, and clomiphene suffered a severe degradation (up to 75%) after 3 days. Such degradation could also take place during the automated 24-h cycle of sample collection and might explain the non-detection of these compounds (see below). At room temperature (20 °C), compounds such as sibutramine and metandienone experienced a significant decrease of 50% after 1 week; 2,4-dinitrophenol after 3 days; and clomiphene, tamoxifen, and metenolone already after 1 day. Unexpectedly, storage at 4 or 20 °C led to higher concentrations than the nominal value for some compounds. This was the case of ephedrine or norephedrine that was found to be stable in this study but strongly degraded in another study with same temperature and pH conditions [20]. A clear explanation was not found, but it could be due to the higher variability in measurements induced by changes in matrix components as a result of storage at higher temperature, or in the case of norephedrine, due to in-sample transformation from amphetamine [21].

**Table 1:** Results of the stability tests conducted at three different temperatures. Results are expressed as the time point (in days) where the change from the initial concentration is higher than 20% for each compound

	20 °C	4 °C	− 20 °C
Norephedrine	30 < 45	> 45	> 45
Ephedrine	0 < 1	7 < 14	> 45
Methylhexanamine	0 < 1	0 < 1	7 < 14
Clenbuterol	30 < 45	30 < 45	> 45
Anastrozole	30 < 45	30 < 45	30 < 45
Sibutramine	1 < 3	14 < 30	30 < 45
Trenbolone	0 < 1	1 < 3	30 < 45
Nandrolone	0 < 1	0 < 1	30 < 45
Metandienone	3 < 7	7 < 14	> 45
Finasteride	0 < 1	3 < 7	> 45
Clomiphene	0 < 1	0 < 1	1 < 3
Mibolerone	> 45	> 45	> 45
Metenolone	0 < 1	0 < 1	> 45
Tamoxifen	0 < 1	0 < 1	> 45
2,4-Dinitrophenol	1 < 3	7 < 14	> 45

#### Method performance

Table 2 summarizes the results of the method validation. The linearity of the response expressed by the regression coefficient (*r*) invariably showed a value above 0.99, without a significant difference between solutions prepared in ultrapure water and wastewater. For the further quantitative analyses, the calibration in ultrapure water was chosen. LODs ranged from 0.2 ng L^−1^ for anastrozole to 20 ng L^−1^ for mibolerone, trenbolone, and nandrolone. LOQs were below 30 ng L^−1^ except for mibolerone, for which only the high concentration level was successfully validated, and trenbolone and nandrolone, for which, depending on the wastewater tested (see ESM Table S4), the LOQs slightly varied around 50 ng L^−1^. The precision, expressed as relative standard deviation (RSD%), remained between 2 and 15%, with the exception of drostanolone (33%). Procedural recoveries were in general satisfactory and ranged from 60 to 130%, with the exception of metenolone, which presented the highest recoveries (up to 160%). For this compound, a deuterated analogue was not available, and this might be the reason for such a high value. RSDs remained below 16% in all cases, which indicated low variability. Matrix effects ranged from 43 to 121%, with RSD up to 22%. Norephedrine, anastrozole, nandrolone, metenolone, and drostanolone seemed to be suppressed by the matrix, whereas the response of ephedrine was slightly enhanced. For clomiphene and tamoxifen, the matrix effect could not be evaluated since no signal was detected in the spiked ultrapure water sample, which corresponds to 100% matrix suppression.

**Table 2:** Method performance in terms of linearity, limits of detection and quantification, intra-day and inter-day precision, procedural recovery, and matrix effects

	Linearity	Limits		Precision				Recovery^c^		Matrix effect^c^	
	*r*	LOD	LOQ	Intra-day RSD (%) (*n* = 6)		Inter-day RSD (%) (*n* = 18, day = 3)		[*γ*/*β*] ± RSD (%) (*n* = 6)		[*β*/*α*] ± RSD (%) (*n* = 6)	
		ng L^−1^	ng L^−1^	50 ng L^−1^	200 ng L^−1^	50 ng L^−1^	200 ng L^−1^	50 ng L^−1^	200 ng L^−1^	50 ng L^−1^	200 ng L^−1^
Norephedrine	0.9997	3	10	4	4	4	3	132 ± 7	84 ± 9	82 ± 9	93 ± 7
Ephedrine	0.9997	3	10	5	2	4	3	125 ± 9	105 ± 9	121 ± 12	89 ± 5
Methylhexanamine^a^	0.9989	7	25	9	6	8	8	63 ± 15	70 ± 4	92 ± 16	87 ± 12
Clenbuterol	0.9987	3	10	4	3	10	10	92 ± 7	69 ± 15	96 ± 7	100 ± 5
Anastrozole	0.9988	0.2	0.7	4	8	7	12	92 ± 8	59 ± 14	74 ± 8	87 ± 9
Sibutramine	0.9997	0.7	2	5	3	8	7	130 ± 11	104 ± 11	64 ± 12	91 ± 22
Trenbolone	0.9923	20	50	4	5	5	9	91 ± 9	70 ± 16	101 ± 7	96 ± 10
Nandrolone	0.9994	20	50	12	11	7	14	104 ± 14	99 ± 15	89 ± 18	77 ± 18
Metandienone	0.9993	7	25	4	2	6	4	105 ± 8	85 ± 11	95 ± 3	96 ± 9
Finasteride	0.9934	0.7	2	2	6	12	13	84 ± 10	61 ± 14	107 ± 14	102 ± 16
Clomiphene	0.9997	7	25	4	2	10	5	96 ± 10	73 ± 14	n.a.	n.a.
Mibolerone^b^	0.9986	20	60	–	3	–	3	–	99 ± 4	–	111 ± 5
Metenolone	0.9986	3	10	4	4	14	14	160 ± 16	147 ± 5	43 ± 10	45 ± 16
Tamoxifen	0.9996	7	25	6	2	8	3	98 ± 9	69 ± 12	n.a.	n.a.
2,4-Dinitrophenol	0.9995	9	30	6	5	13	7	77 ± 16	60 ± 20	65 ± 7	88 ± 5

LOD and LOQ obtained for pooled wastewater sample. Table SI-4 presents LODs and LOQs specific for each of the sampling locations

*n*.*a*. not available, compound non-detected in ultrapure water (*α*), equivalent to 100% matrix suppression

^a^*n* = 5

^b^Only one level was successfully validated for this compound

^c^For explanation of symbols used in recovery and matrix effect calculations, see the text

### Application to wastewater samples

#### Local wastewater characteristics

As already highlighted above, wastewater is a complex matrix. The method validation presented in the previous section corresponds to the performance obtained with an in-house reference sample made from pooled wastewater. However, the composition of wastewater is very much affected by geographical location and temporal characteristics. For this reason, the procedural recovery and the limits of detection and quantification were re-evaluated for each location sampled. To this end, with every sample batch, three samples were analyzed in duplicate one being spiked at 200 ng L^−1^. Results are presented in Table S4 (see the ESM).

The variability of the recoveries remained below 25% in all but three cases. Ephedrine showed high variability in the samples from WWTPs A and C due to higher noise signals in the chromatograms. Regarding the limits of detection and quantification, in general, they were in line (similar range) with those obtained with the reference-pooled wastewater, although some differences were observed. In the case of methylhexanamine and clenbuterol, lower LOQs were observed at all locations, whereas for ephedrine, anatrozole, finasteride, mibolerone, metenolone, clomiphene, tamoxifen, and drostanolone LOQs were higher at all locations. For the remaining compounds (norephedrine, sibutramine, trenbolone, nandrolone, and metadienone) LOQs were in the same range as those previously estimated in the reference-pooled wastewater.

#### Results obtained for the wastewater influents

The concentrations calculated in wastewater influents were transformed into influent loads (expressed in mg day^−1^, see Tables S5 to S8 in the ESM) by multiplying with the daily influent flow rates provided by the WWTP operators (presented in ESM Table S3).

##### Event A

The results obtained for the samples collected at the entrance of the WWTP A (ESM Table S5) showed the presence of three weight loss compounds in all the samples: ephedrine with the highest loads, ranging from 70 to 122 g day^−1^, followed by norephedrine, ranging from 6.6 to 14 g day^−1^, and methylhexanamine, ranging from 7 to 13 g day^−1^. From the other weight loss products, sibutramine, was quantified in 4 out of the 15 samples analyzed, ranging from 776 to 2910 mg day^−1^, and detected in other 4 samples < LOQ, and 2,4-dinitrophenol was quantified in 1 sample at 4300 mg day^−1^ and detected in other 1 sample < LOQ. The anabolic steroid metandienone was quantified in 5 out of the 15 samples, ranging from 1480 to 2020 mg day^−1^, and detected in other 8 samples < LOQ. Clenbuterol was detected in 1 sample < LOQ. None of the remaining compounds were detected (i.e., no signal or signal below LOD).

Statistical evaluation of the loads during the event and the corresponding reference weekend using two-tailed Mann-Whitney test (*p* < 0.05 significant difference) showed that the medians of both periods were significantly different for norephedrine (*p* = 0.03). This suggested an enhanced use of norephedrine during the event weekend. This stimulant is most commonly used to reduce body fat before a sport event by fast increasing human metabolism. However, it is also a metabolite of the recreational substance amphetamine [21], and therefore, the increase could also be caused by an enhanced consumption of this illegal substance (and its degradation from wastewater [20]).

##### Event B

The results obtained for the samples collected at the entrance of the WWTP B (ESM Table S6) showed the presence of three weight loss compounds in all the samples: ephedrine with the highest loads, ranging from 6 to 15 g day^−1^, followed by norephedrine (457 to 894 mg day^−1^), and methylhexanamine (254 to 710 mg day^−1^). From the other weight loss products, sibutramine was quantified in 1 out of the 15 samples analyzed, at 90 mg day^−1^, and was detected, albeit below LOQ, in another 10 samples, and 2,4-dinitrophenol was quantified in 3 samples ranging from 516 to 6430 mg day^−1^ and present at levels between LOD and LOQ in another 6 samples. The anabolic steroid metandienone was detected in all of the samples at levels between LOD and LOQ.

An increase in loads just prior to the event weekend can be observed for ephedrine, norephedrine, methylhexanamine, and 2,4-dinitrophenol. This increase in loads might be explained by the fact that the athletes arrived already on Friday or Saturday. Statistically evaluating the use of ephedrine, norephedrine, and methylhexanamine in the two weekends (and the days prior to the weekend) using a two-tailed Mann-Whitney test, a significant difference between the medians was observed for norephedrine and methylhexanamine (*p* = 0.03 in both cases) but not for ephedrine. In the case of 2,4-dinitrophenol, there was not sufficient data for a statistical evaluation. However, its use just prior to and during the sport event was obvious as it was found to be present at quantifiable concentrations only in those days (ESM Table S6), while being < LOQ during the reference weekend (see also section “Normalized loads” below). We conclude that weight loss products had been used on the days prior to the sport event B. The case of 2,4-dinitrophenol was particularly noteworthy, because its use has been associated with severe adverse effects and even risk of death [18].

##### Event C

The results obtained for the samples collected at the pumping station C and the entrance of the WWTP C (ESM, Tables S7 and S8) revealed again the presence of three weight loss compounds in all of the samples. The highest loads were observed for ephedrine, ranging from 4 to 33 g day^−1^ at the pumping station and 29 to 108 g day^−1^ at the WWTP entrance, respectively. Norephedrine loads ranged from 341 to 1400 and 4540 to 8790 mg day^−1^, respectively, and methylhexanamine loads from 594 to 2020 and 4450 to 125,000 mg day^−1^, respectively. From the other weight loss products, sibutramine was quantified in 6 out of the 14 samples analyzed in wastewater from pumping station C, ranging from 22 to 335 mg day^−1^, and detected in 2 more samples at a level between LOD and LOQ. In the influent of WWTP C, sibutramine was quantified in 5 out of the 16 samples analyzed, ranging from 295 to 1660 mg day^−1^, and detected in 6 more samples at concentrations between LOD and LOQ. 2,4-Dinitrophenol was quantified in 7 out of the 14 samples analyzed in wastewater from pumping station C, ranging from 255 to 9740 mg day^−1^, and detected in 5 more samples at a level between LOD and LOQ, and in the case of WWTP C 2,4-DNP was quantified in 6 out of the 16 influent samples analyzed, ranging from 6390 to 717,000 mg day^−1^, and detected in 10 more samples at a level between LOD and LOQ. Metandienone was quantified in 4 out of 16 samples from the influent of WWTC C, ranging from 761 to 1190 mg day^−1^. Clenbuterol, clomiphene, and nandrolone were just detected at levels between LOD and LOQ (clenbuterol, 1 sample at each location; clomiphene, 2 samples from pumping station C; nandrolone, 2 samples from WWTP C).

Statistical evaluation of the loads during the event and the corresponding reference weekend using two-tailed Mann-Whitney test (*p* < 0.05 significant difference) showed that the medians of both periods and both locations were not significantly different for ephedrine, norephedrine, and methylhexanamine. Once again in the case of 2,4-dinitrophenol, there was not sufficient data for a statistical evaluation; however, its use just prior to and during the sport event was obvious (at both locations) due to the relatively high levels quantified during those days, while loads were much lower or below LOQs in the reference weekend (see also the next section).

#### Normalized loads

**Fig. 1:**
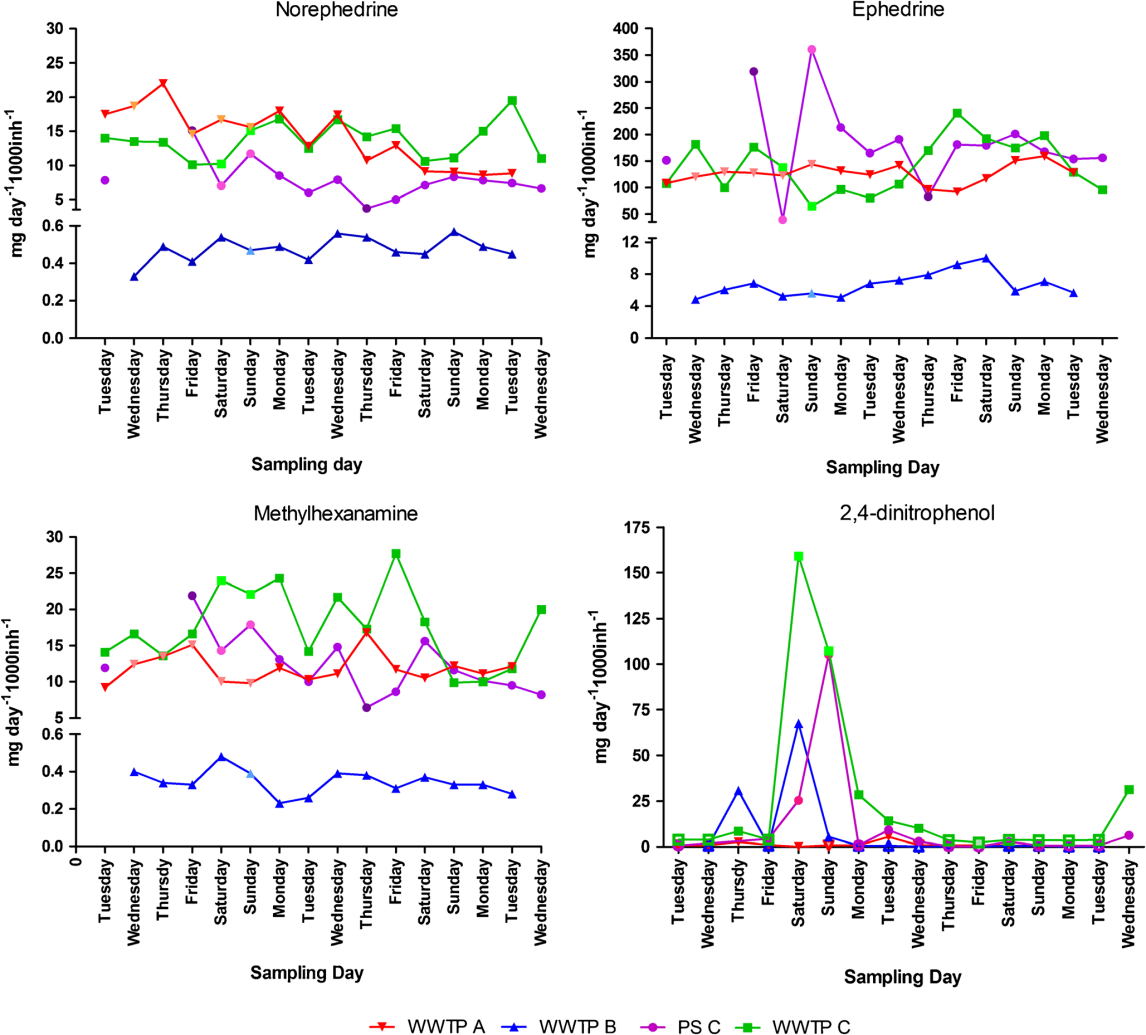
Normalized loads (expressed in mg day^−1^ 1000 inh^−1^) of the most detected weight loss products (ephedrine, norephedrine, methylhexanamine, and 2,4-dinitrophenol) per sampling location

The comparison of the normalized loads per compound is presented in Fig. 1. In the case of norephedrine, ephedrine, and methylhexanamine, the trend is similar. Lower normalized loads correspond to the small-size city sampled at WWTP B. In the large and medium-size cities (A and C), the normalized loads appear to be similar. One characteristic that might support this difference between A and C against B is the relative number of students in each of the cities. Whereas in A and C, the percentage of residing students represents a 14% of the total population, in B it only represents 0.2% [22]. In the past, many studies have revealed the use of stimulant drugs by college students for cognitive enhancement, specially the use of methylphenidate, the active pharmaceutical ingredient for the treatment of attention deficit hyperactivity disorder, but also other type of prescription and lifestyle stimulant drugs. A recent study in the Netherlands showed evidence of polydrug use with this purpose, although with lower prevalence than in other countries in Europe (1.6 versus 4.6–16%) [23].

It is noteworthy that the normalized loads from the pumping station, which correspond to a small part (20%) of the larger city C, also match with the larger cities (A and C). This might be explained by the effect of the sport event. Whereas event A was held for professional athletes and official anti-doping controls were performed, events B and C were held for amateur athletes and no anti-doping measures were taken. Especially event C is also known among the amateur bodybuilding community, and not only the participants might use illicit substances but also the event visitors.

The normalized loads of 2,4-dinitrophenol clearly reveal an aberrantly high load during the B and C events. This compound was originally used in the manufacture of dyes, wood preservatives, and as a pesticide. However, another use was discovered as solution for rapid weight loss. Although it seems an effective solution to this end, it is highly toxic, and even small overdoses have been reported to result in death [24, 25]. The high loads found during the events (including the day before the events start, but not before, and not after) might indicate the use as weight loss, since such a sudden increase/decrease would not be expected in any other of its known uses, for example as a pesticide. It is therefore of great concern to discover its use, and measures should be taken to inform and bring awareness to the athletes community, not only restricted to anti-doping.

Finally, an interesting finding is the relative absence of detection of analytes from the group of anabolic steroids. However, it is important to stress that the targeted analytes in this method are the parent compounds, and they might not be the best biomarkers due to excretion as conjugates or in the form of metabolites or transformation products. Temperatures of the wastewaters were such that for a few compounds some degradation may have occurred in-sewer (see Table 1 and ESM Table S3). Therefore, estimation of the use of these compounds in wastewater would require a more in-depth study on the most suitable biomarkers to be determined in this complex matrix.

## Conclusions

Chemical analysis of wastewater can reveal the use of doping agents by the general population and during sport events. The results of the present study provided valuable information that can be of interest for anti-doping authorities. The analytical methodology developed in this work, based on the use of LC-MS/MS, allowed the detection and quantification in wastewater of doping substances used by the general population and amateur athletes attending the targeted events. Weight loss stimulants, namely ephedrine, norephedrine, and methylhexanamine, were found in high amounts. In addition the detection of 2,4-dinitrophenol is a major concern due to its known adverse effects. The results suggested the increase in loads of some substances possibly during the monitored sport events.

Further refinement of analysis to include metabolites and transformation products would provide valuable information, especially in the cases of compounds rapidly metabolized by the human body and therefore not present in the urine and wastewater (or present in minor quantities).

## Electronic supplementary material

ESM 1 (PDF 1028 kb)
