# Supplementary material for: Wastewater-based tracing of doping use by the general population and amateur athletes
Source: Anal Bioanal Chem. 2018 Jan 15;410(6):1793–803. doi: 10.1007/s00216-017-0835-3 (PMC5807464; doi:10.1007/s00216-017-0835-3)
Supplement: Supplementary file 1 — (PDF 1028 kb) [file 216_2017_835_MOESM1_ESM.pdf]

Analytical and Bioanalytical Chemistry

Electronic Supplementary Material

Wastewater-based tracing of doping use by the general population and amateur athletes

Ana Causanilles, Vera Nordmann, Dennis Vughs, Erik Emke, Olivier de Hon,
Félix Hernández, Pim de Voogt

Table S1 List of the selected target compounds with their chemical properties description

Name	Type of action	ATC code	CAS number	Formula	Log Kow	pKa[1]	Structure
Metandienone	anabolic-androgenic steroid	A14AA03	72-63-9	$C_{20}H_{28}O_2$	3.51	-0.53 - 14.53	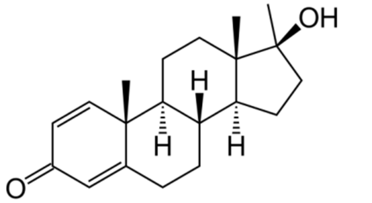
Metenolone	anabolic-androgenic steroid	A14AA04	153-00-4	$C_{20}H_{30}O_2$	3.69	-0.88 – 19.38	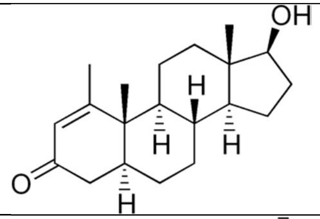
Mibolerone	anabolic-androgenic steroid	None	3704-09-4	$C_{20}H_{30}O_2$	3.19	-0.53 - n.a.	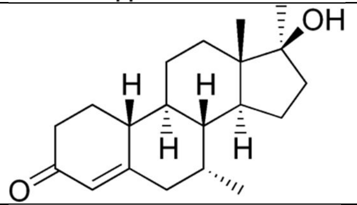
Nandrolone	anabolic-androgenic steroid	A14AB01 S01XA11	434-22-0	$C_{18}H_{26}O_2$	2.62	-0.88 – 19.28	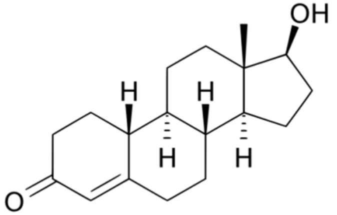

Trenbolone	anabolic-androgenic steroid	none	10161-33-8	$C_{18}H_{22}O_2$	2.65	-0.89 – n.a.	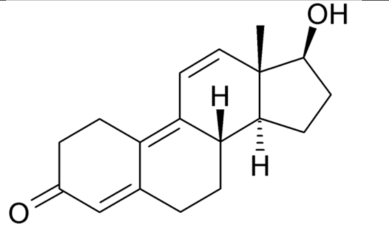
Clenbuterol	β2 agonist – performance-enhancing drug, also weight-loss	R03AC14 R03CC13	37148-27-9	$C_{12}H_{18}Cl_2N_2O$	2.00	9.63 – 14.06	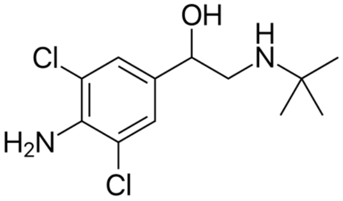
Ephedrine	Weight-loss – substitited phenethylamine stimulant	C01CA26	299-42-3	$C_{10}H_{15}NO$	1.13	9.52 – 13.89	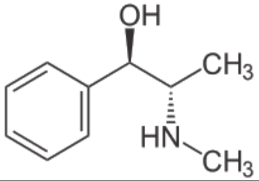
Norephedrine	Weight-loss – substitited phenethylamine stimulant	none	14838-15-4	$C_9H_{13}NO$	0.67	9.37 – 13.90	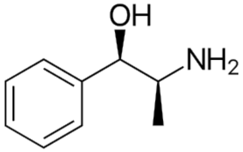
Methylhexaneamine	Weight-loss - sympathomimetic drug	none	105-41-9	$C_7H_{17}N$	2.16	10.54 – n.a.	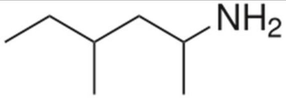
Sibutramine	Weight-loss – serotonin-norepinephrine reuptake inhibitor	A08AA10	106650-56-0	$C_{17}H_{26}NCl$	5.73	9.77 – n.a.	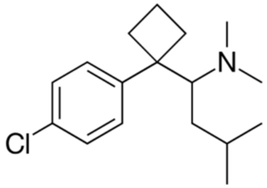

2,4-dinitrophenol	Weight-loss	none	51-28-5	$C_6H_4N_2O_5$	1.67	-7.80 – 4.04	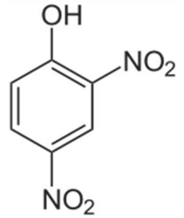
Clomiphene	hormone antagonist - antiestrogen	G03GB02	911-45-5	$C_{26}H_{28}ClNO$	6.74	9.31 – n.a.	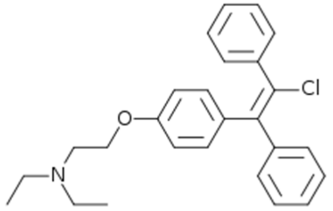
Tamoxifen	hormone antagonist - antiestrogen	L02BA01	10540-29-1	$C_{26}H_{29}NO$	6.30	8.76 – n.a.	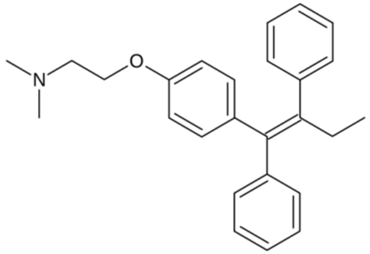
Anastrozole	hormone antagonist - antiestrogen	L02BG03	120511-73-1	$C_{17}H_{19}N_5$	2.37	2.25 – n.a.	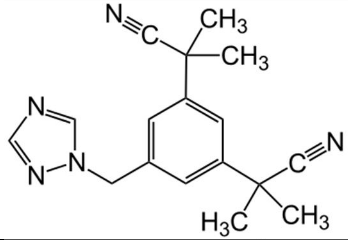
Finasteride	hormone antagonist - 5-α-reductase-inhibitor	D11AX10 G04CB01	98319-26-7	$C_{23}H_{36}N_2O_3$	3.03	2.22 – 14.53	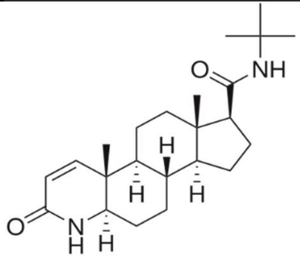

[1] Strongest basic – strongest acidic (ChemAxon)
None: not available or not existing

Table S2 LC-HRMS acquisition parameters: t_R, $[M+H]^+$ (*except $[M-H]^-$ for 2,4-dinitrophenol), optimal collision energy (CE), and accurate mass for at least one product ion

	t_R (min)	$[M+H]^+$	CE	$[M+H]^+$ (product ion 1)	$[M+H]^+$ (product ion 2)	ILIS used
Norephedrine	4.1	152.1070	40	91.0539	115.0536	Norephedrine-d_3
Ephedrine	4.5	166.1226	50	91.0538	115.0532	Ephedrine-d_3
Methylhexanamine	6.3	116.1434	15	57.0699	—	Methylhexanamine-d_4
Clenbuterol	6.6	277.0869	30	203,0132	168,0441	Clenbuterol-d_9
Anastrozole	8.8	294.1713	25	225,1388	210.1150	Anastrozole-d_{12}
Sibutramine	10.5	280.1827	25	125.0145	139.0301	Sibutramine-d6
Trenbolone	11.2	271.1693	25	199.1115	253.1594	Trenbolone-d_5
Nandrolone	11.5	275.2006	32	109.0635	145.0997	Methandrostenolone-d_3
Metandienone	11.7	301.2162	25	121.0632	149.1308	Methandrostenolone-d_3
Finasteride	11.9	373.2850	32	317.2237	305.2598	Finasteride-d_9
Clomiphene	12.0	406.1932	30	100.1113	297.1278	Clomiphene-d_5
Mibolerone	12.3	303.2319	30	285.2197	121.0992	Mibolerone-d_3
Metenolone	12.5	303.2319	30	187.1472	83.0482	Mibolerone-d_3
Tamoxifen	12.4	372.2322	35	72.0807	129.0690	Tamoxifen-d_5
2,4-dinitrophenol	8.7	183.0047*	-30	123.0440*	—	2,4-dinitrophenol-d_3

Table S2 (continued) LC-HRMS acquisition parameters: t_R, $[M+H]^+$ (*except $[M-H]^-$ for 2,4-dinitrophenol), optimal collision energy (CE), and accurate mass for at least one product ion

**ILIS**	**t_R (min)**	**$[M+H]^+$**	**CE**	**$[M+H]^+$ (product ion 1)**	**$[M+H]^+$ (product ion 2)**
Norephedrine-d_3	4.1	155.1258	35	93.0663	117.0662
Ephedrine-d_3	4.5	169.1415	30	136.1067	117.0692
Methylhexanamine-d_4	6.2	120.1685	15	—	—
Clenbuterol-d_9	6.5	286.1434	30	204.0178	169.0487
Anastrozole-d_{12}	8.7	306.2466	25	237.2123	—
Sibutramine-d_6	10.5	286.2203	25	179.0602	153.0446
Trenbolone-d_5	11.1	276.2006	25	258.1879	204.1406
Methandrostenolone-d_3	11.7	304.2350	25	121.0628	152.1491
Finasteride-d_9	11.8	382.3414	32	317.2237	305.2598
Clomiphene-d_5	12.0	411.2246	30	100.1103	302.1567
Mibolerone-d_3	12.2	306.2507	30	288.2385	124.1180
Tamoxifen-d_5	12.4	377.2636	35	72.0805	70.0645
2,4-dinitrophenol-d_3	8.6	186.0236*	-30	126.0270*	—

Table S3 Sample weekday and corresponding daily influent flow rates at the different sampling locations

		Flow m^3 d^{-1}			
		WWTP A	WWTP B	Event C	
				Pumping station[3]	WWTP C
Number of inhabitants		769,000	95,000	92,500	450,300
Range of temperatures		18 – 25 °C	2 – 10 °C	5 – 12 °C	5 – 12 °C
Sample day	Tuesday	155546	-	14439	96823
	Wednesday	**155708**	11590	14633[2]	98325
	Thursday	**148062**	23512	15047[2]	100873
	Friday	**156895**	n.a.[1]	27527	97194
	Saturday	**144113**	22759	**14040**	**94546**
	Sunday	**142268**	**14933**	**24811**	**136543**
	Monday	144487	12194	17032	114614
	Tuesday	150896	11415	14283	100593
	Wednesday	151886	11439	20023	126486
	Thursday	140841	11400	7900	90095
	Friday	141184	11360	11473	105499
	Saturday	134028	11833	14699	94348
	Sunday	135930	10810	14341	89267
	Monday	135967	10478	13606	87453
	Tuesday	134850	12282	14563	94966
	Wednesday	-	12239	14033	96754

Values in bold correspond to the event days.

[1] n.a. not available due to pump failure.

[2] Autosampler failed and no sample was collected.

[3] At the pumping station flow rate measurements are most reliable when between 7,000 and 15,000 m^3 (dry weather days). At higher flow rates, caused by heavy rainfall, a bypass is switched on to handle the incoming influent. Flow rates from these days may therefore be underestimated. Notwithstanding, the actual flow rates given by the WWTP operators were used, as no other flow data were available and will provide minimum loads. As the number of days with such events was only minor (3) this will be of little influence on the total picture.

Table S4 Results from the re-evaluation of the method performance with spiked wastewater samples from each sampling location in terms of recovery, R (%) and relative standard deviation (RSD, %), limits of detection (LOD) and quantification (LOQ)

	WWTP A			WWTP B			Pumping station C			WWTP C		
	R ± RSD (%)[1]	LOD (ng L^{-1})	LOQ (ng L^{-1})	R (%)[2]	LOD (ng L^{-1})	LOQ (ng L^{-1})	R ± RSD (%)[1]	LOD (ng L^{-1})	LOQ (ng L^{-1})	R ± RSD (%)[1]	LOD (ng L^{-1})	LOQ (ng L^{-1})
Norephedrine	98 ± 14	4	14	106	3	9	98 ± 2	2	6	94 ± 15	1	4
Ephedrine	43 ± 94	4	12	28	15	49	160 ± 19	5	18	20 ± 54	5	16
Methylhexanamine	100 ± 5	4	14	102	5	16	85 ± 10	4	14	131 ± 4	7	22
Clenbuterol	89 ± 6	2	8	96	2	6	81 ± 2	2	7	87 ± 3	2	6
Anastrozole	89 ± 7	1	4	78	0	1	86 ± 12	1	4	90 ± 3	1	4
Sibutramine	88 ± 7	1	3	133	1	5	98 ± 12	0.5	2	89 ± 3	1	4
Trenbolone	112 ± 7	16	54	106	11	36	135 ± 2	13	44	102 ± 2	20	68
Nandrolone	29 ± 11	3	12	66	40	135	18 ± 20	12	41	17 ± 4	22	72
Metadienone	97 ± 9	3	10	104	12	42	105 ± 6	3	10	107 ± 3	3	8
Finasteride	104 ± 17	25	82	112	2	6	125 ± 22	19	64	170 ± 4	19	62
Clomiphene	98 ± 5	18	61	110	37	128	84 ± 8	26	85	92 ± 15	25	84
Mibolerone	110 ± 11	33	111	116	48	160	112 ± 4	55	182	127 ± 3	49	165
Metenolone	13 ± 21	6	20	83	23	78	12 ± 28	11	36	12 ± 22	15	51
Tamoxifen	103 ± 7	31	103	106	24	81	88 ± 14	37	124	94 ± 22	48	161
2,4-dinitrophenol[3]	95	9	28	88	3	9	95	3	9	117	11	36

[1] n=3
[2] n=1, no RSD(%)
[3] n=1, no RSD(%)

Table S5 Loads of doping substances expressed in mg d^{-1} quantified in the influent samples collected at the WWTP A

Sample day	Norephedrine	Ephedrine	Methylhexanamine	Clenbuterol	Anastrozole	Sibutramine	Trenbolone	Nandrolone
Tuesday	13400	83200	7040	—	—	1090	—	—
**Wednesday**	**14400**	**92400**	**9530**	+	—	**2910**	—	—
**Thursday**	**15400**	**99600**	**10400**	—	—	+	—	—
**Friday**	**11300**	**98000**	**11600**	—	—	—	—	—
**Saturday**	**12900**	**94200**	**7680**	—	—	+	—	—
**Sunday**	**12000**	**111000**	**7560**	—	—	—	—	—
Monday	13900	101000	9160	—	—	—	—	—
Tuesday	9850	95500	7930	—	—	—	—	—
Wednesday	13400	109000	8520	—	—	776	—	—
Thursday	8260	74200	12900	—	—	+	—	—
Friday	9890	70700	9020	—	—	+	—	—
Saturday	7000	90100	8050	—	—	—	—	—
Sunday	6910	116000	9360	—	—	—	—	—
Monday	6610	122000	8530	—	—	—	—	—
Tuesday	6740	98700	9310	—	—	1270	—	—

— below LOD
+ below LOQ

Values in bold correspond to the event days.

Table S5 (continued) Loads of doping substances expressed in mg d^{-1} quantified in the influent samples collected at the WWTP A

Sample day	Metandienone	Finasteride	Clomiphene	Mibolerone	Metenolone	Tamoxifen	2,4-dinitrophenol
Tuesday	+	—	—	—	—	—	—
**Wednesday**	**2020**	—	—	—	—	—	—
**Thursday**	+	—	—	—	—	—	+
**Friday**	+	—	—	—	—	—	—
**Saturday**	**1480**	—	—	—	—	—	—
**Sunday**	**1700**	—	—	—	—	—	—
Monday	1600	—	—	—	—	—	—
Tuesday	—	—	—	—	—	—	4300
Wednesday	—	—	—	—	—	—	—
Thursday	+	—	—	—	—	—	—
Friday	1490	—	—	—	—	—	—
Saturday	+	—	—	—	—	—	—
Sunday	+	—	—	—	—	—	—
Monday	+	—	—	—	—	—	—
Tuesday	+	—	—	—	—	—	—

— below LOD
+ below LOQ

Values in bold correspond to the event days.

Table S6 Loads of doping substances expressed in mg d^{-1} quantified in the influent samples collected at the WWTP B

Sample day	Norephedrine	Ephedrine	Methylhexanamine	Clenbuterol	Anastrozole	Sibutramine	Trenbolone	Nandrolone
Wednesday	474	6530	478	—	—	+	—	—
Thursday	730	10800	904	—	—	+	—	—
Friday	658	8130	453	—	—	+	—	—
Saturday	894	14800	710	—	—	+	—	—
**Sunday**	**764**	**7400**	**676**	**—**	**—**	**+**	**—**	**—**
Monday	543	6480	455	—	—	+	—	—
Tuesday	526	5520	254	—	—	—	—	—
Wednesday	462	7410	279	—	—	—	—	—
Thursday	609	7860	421	—	—	90	—	—
Friday	580	8550	412	—	—	+	—	—
Saturday	519	10400	352	—	—	+	—	—
Sunday	457	10300	384	—	—	+	—	—
Monday	565	5850	332	—	—	+	—	—
Tuesday	567	8240	384	—	—	—	—	—
Wednesday	474	6530	478	—	—	—	—	—

— below LOD
+ below LOQ

Values in bold correspond to the event days.

Table S6 (continued) Loads of doping substances expressed in mg d^{-1} quantified in the influent samples collected at the WWTP B

Sample day	Metandienone	Finasteride	Clomiphene	Mibolerone	Metenolone	Tamoxifen	2,4-dinitrophenol
Wednesday	+	—	—	—	—	—	+
Thursday	+	—	—	—	—	—	2930
Friday	+	—	—	—	—	—	+
Saturday	+	—	—	—	—	—	6430
**Sunday**	**+**	**—**	**—**	**—**	**—**	**—**	**516**
Monday	+	—	—	—	—	—	+
Tuesday	+	—	—	—	—	—	+
Wednesday	+	—	—	—	—	—	—
Thursday	+	—	—	—	—	—	—
Friday	+	—	—	—	—	—	—
Saturday	+	—	—	—	—	—	+
Sunday	+	—	—	—	—	—	+
Monday	+	—	—	—	—	—	—
Tuesday	+	—	—	—	—	—	—
Wednesday	+	—	—	—	—	—	—

— below LOD
+ below LOQ

Values in bold correspond to the event days.

Table S7 Loads of doping substances expressed in mg d^{-1} quantified in the influent samples collected at the pumping station C

	Norephedrine	Ephedrine	Methylhexanamine	Clenbuterol	Anastrozole	Sibutramine	Trenbolone	Nandrolone
Tuesday	721	14000	1100	+	—	335	—	—
Friday	1400	29500	2020	—	—	70	—	—
**Saturday**	**651**	**3630**	**1320**	**—**	**—**	**22**	**—**	**—**
**Sunday**	**1080**	**33300**	**1660**	**—**	**—**	**+**	**—**	**—**
Monday	791	19700	1210	—	—	35	—	—
Tuesday	553	15300	926	—	—	—	—	—
Wednesday	727	17700	1370	—	—	+	—	—
Thursday	341	7650	594	—	—	—	—	—
Friday	463	16700	796	—	—	—	—	—
Saturday	655	16600	1440	—	—	—	—	—
Sunday	767	18600	1070	—	—	—	—	—
Monday	722	15500	936	—	—	—	—	—
Tuesday	685	14200	880	—	—	41	—	—
Wednesday	609	14400	755	—	—	78	—	—

— below LOD
+ below LOQ

Values in bold correspond to the event days.

Table S7 (continued) Loads of doping substances expressed in mg d^{-1} quantified in the influent samples collected at the pumping station C

	Metandienone	Finasteride	Clomiphene	Mibolerone	Metenolone	Tamoxifen	2,4-dinitrophenol
Tuesday	—	—	+	—	—	—	+
Friday	—	—	—	—	—	—	397
**Saturday**	**—**	**—**	**—**	**—**	**—**	**—**	**2350**
**Sunday**	**—**	**—**	**—**	**—**	**—**	**—**	**9740**
Monday	—	—	—	—	—	—	+
Tuesday	—	—	—	—	—	—	849
Wednesday	—	—	—	—	—	—	273
Thursday	—	—	—	—	—	—	+
Friday	—	—	—	—	—	—	—
Saturday	—	—	—	—	—	—	255
Sunday	—	—	—	—	—	—	—
Monday	—	—	—	—	—	—	+
Tuesday	—	—	—	—	—	—	+
Wednesday	—	—	+	—	—	—	581

— below LOD
+ below LOQ

Values in bold correspond to the event days.

Table S8 Loads of doping substances expressed in mg d^{-1} quantified in the influent samples collected at the WWTP C

Sample day	Norephedrine	Ephedrine	Methylhexanamine	Clenbuterol	Anastrozole	Sibutramine	Trenbolone	Nandrolone
Tuesday	6320	48300	6340	—	—	468	—	—
Wednesday	6090	81800	7460	—	—	295	—	—
Thursday	6050	44800	6110	—	—	+	—	—
Friday	4540	79400	7480	—	—	+	—	—
**Saturday**	**4590**	**62100**	**10800**	**—**	**—**	**+**	**—**	**—**
**Sunday**	**6820**	**29300**	**9960**	**—**	**—**	**+**	**—**	**+**
Monday	7550	43500	11000	—	—	+	—	+
Tuesday	5610	36200	6380	—	—	—	—	—
Wednesday	7540	47800	9760	—	—	—	—	—
Thursday	6400	76600	7780	—	—	+	—	—
Friday	6930	108000	12500	—	—	—	—	—
Saturday	4760	86600	8250	—	—	—	—	—
Sunday	4980	78700	4450	—	—	—	—	—
Monday	6750	89300	4520	—	—	776	—	—
Tuesday	8790	58100	5330	+	—	1660	—	—
Wednesday	4960	43100	9020	—	—	355	—	—

— below LOD
+ below LOQ

Values in bold correspond to the event days.

Table S8 (continued) Loads of doping substances expressed in mg d^{-1} quantified in the influent samples collected at the WWTP C

Sample day	Metandienone	Finasteride	Clomiphene	Mibolerone	Metenolone	Tamoxifen	2,4-dinitrophenol
Tuesday	1190	—	—	—	—	—	+
Wednesday	+	—	—	—	—	—	+
Thursday	761	—	—	—	—	—	3870
Friday	+	—	—	—	—	—	+
**Saturday**	**+**	**—**	**—**	**—**	**—**	**—**	**71700**
**Sunday**	**+**	**—**	**—**	**—**	**—**	**—**	**48300**
Monday	+	—	—	—	—	—	12800
Tuesday	—	—	—	—	—	—	6390
Wednesday	1050	—	—	—	—	—	+
Thursday	+	—	—	—	—	—	+
Friday	—	—	—	—	—	—	—
Saturday	—	—	—	—	—	—	+
Sunday	—	—	—	—	—	—	+
Monday	—	—	—	—	—	—	+
Tuesday	1130	—	—	—	—	—	+
Wednesday	—	—	—	—	—	—	14100

— below LOD
+ below LOQ

Values in bold correspond to the event days.

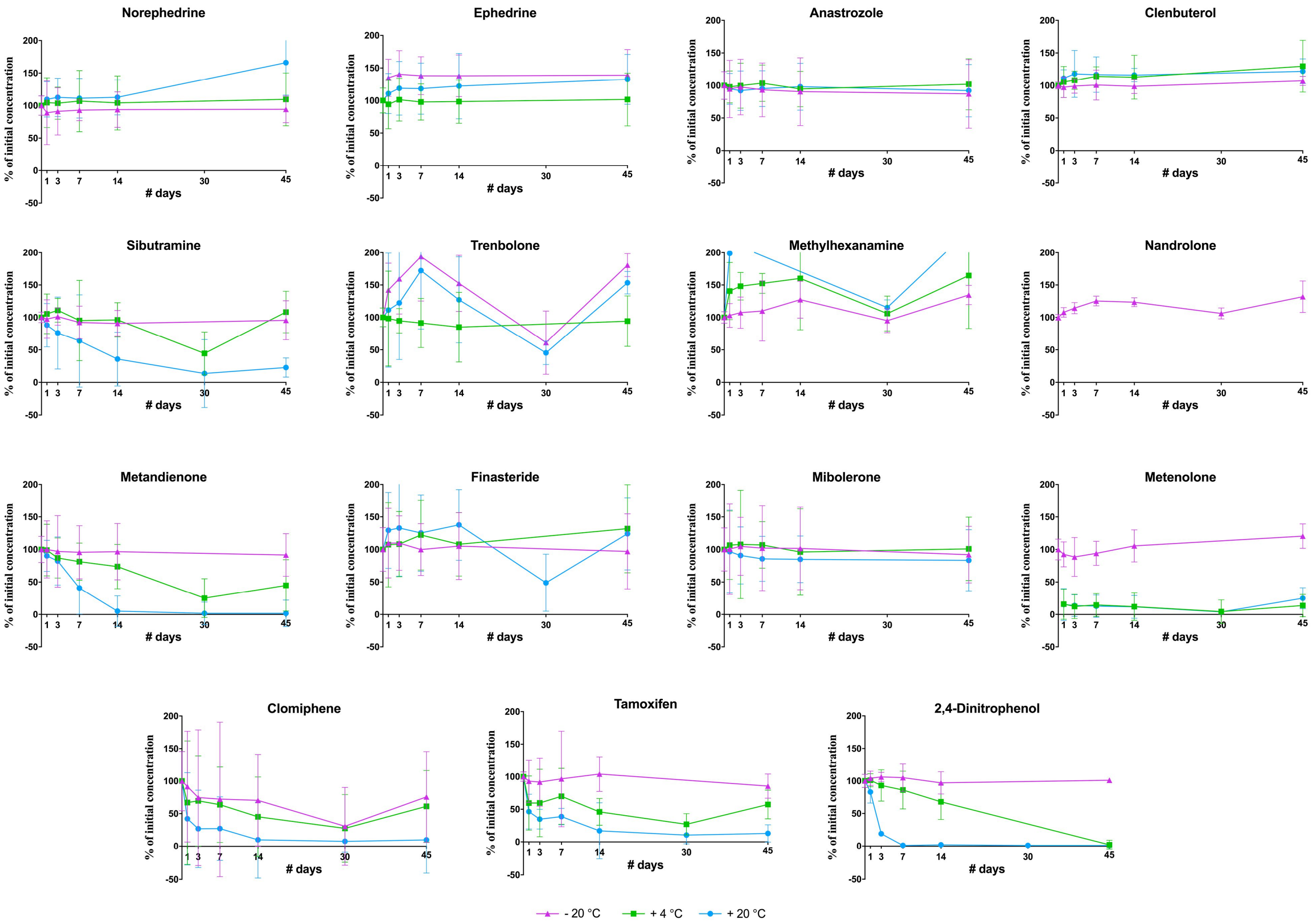

Fig. S1 Stability plots for 15 of the studied compounds in wastewater at natural pH and 3 different temperatures. Results have been normalized as percentage of initial concentration. Error bars correspond to the standard deviation of triplicates
